# Should a Man with Gleet Marry?

**Published:** 1875-01

**Authors:** 


Should a Man with Gleet Marry?—In our opinion, *he should not.* As long as there is the least discharge, sexual intercourse is very apt to irritate the urethra, and bring on a subacute attack. In that condition he could infect his wife. Professor Van Buren says, in his late work on the Veneral (p. 50,) "Gleet is contagious when purulent; the more copious and creamy the discharge, the greater its infecting power."—*Medical and Surgical Reporter.*

